# School bullying victimization, depression, and the role of school connectedness among junior high school students in Hong Kong: evidence from fixed-effects models

**DOI:** 10.1186/s40359-026-04614-2

**Published:** 2026-04-25

**Authors:** Yang Han, Ji-Kang Chen

**Affiliations:** Department of Social Work, The Chinese University of Hong Kong, Shatin, New Territories, Hong Kong, China

**Keywords:** School bullying victimization, Depression, School connectedness, Mediating effect, Fixed-effects models, Hong Kong

## Abstract

**Background:**

School bullying victimization can lead to severe health consequences. However, less is known about the moderating and mediating factors of the association between school bullying victimization and depression, especially in Hong Kong. Therefore, we examined the moderating and mediating roles of school connectedness on the association between school bullying victimization and depression while controlling for unobserved time-invariant confounders among junior high school students in Hong Kong.

**Methods:**

We employed two-wave follow-up data collected from junior high school students (Grades 7–9) in Hong Kong. We used the depression dimension of the Brief Symptom Rating Scale, the California School Climate and Safety Survey, and five items from the National Longitudinal Study of Adolescent Health to measure depression, school bullying victimization, and school connectedness. We conducted fixed-effects linear regression and mediation analysis to answer the research questions.

**Results:**

School bullying victimization was associated with reduced school connectedness (*β* = -0.080, 95% CI: -0.138, -0.021), and reduced school connectedness was associated with greater depression (*β* = -0.049, 95% CI: -0.078, -0.021). School connectedness mediated 12.86% of the association between school bullying victimization and depression. The moderating effect of school connectedness on the association between school bullying victimization and depression was not significant (*β* = -0.002, 95% CI: -0.005,0.001).

**Conclusions:**

Interventions aimed at alleviating depression of students who experienced bullying should assist them in reestablishing their school connectedness. When reestablishing school connectedness to cope with the detrimental impact of bullying victimization on depression, interventions should consider the overall school environment.

**Supplementary Information:**

The online version contains supplementary material available at 10.1186/s40359-026-04614-2.

## Introduction

School bullying has become a global concern. Globally, 32% of students experienced peer bullying in school in the past month [1]. The estimated prevalence of school bullying victimization in Chinese communities was 20.8% [2]. In Hong Kong, approximately 10% of schoolgirls and 25% of schoolboys reported experiencing bullying several times a month [3]. Furthermore, school bullying victimization can cause severe health consequences [4–6].

Although the association between school bullying victimization and ill health are well established, studies on the moderating (namely, the buffering effect) and mediating factors of the association between school bullying victimization and depression are scarce, especially in places such as Hong Kong, where people are influenced by Western and Chinese cultures. Also, although previous studies utilizing cross-sectional designs have significantly enhanced our understanding of school bullying and mental health, results from these studies are unavoidably subject to confounding bias. Hence, considering school connectedness to be a potential buffering and mediating factor in the association between school bullying victimization and depression, we aimed to examine both the moderating and mediating roles of school connectedness in the association between school bullying victimization and depression among junior high school students in Hong Kong. We employed fixed-effects models to eliminate the effects of all the observed and unobserved time-invariant confounders on the associations between school bullying victimization, school connectedness, and depression.

### School bullying victimization and depression

Adolescents’ mental health problems have become a global public health concern. It was estimated that 14% of adolescents aged 10–19 years old have mental health conditions worldwide [7]. Depression is one of the common mental health disorders among adolescents. Globally, approximately 20% of youth under 18 years old suffered from depression, and the prevalence increased over time [8]. In Hong Kong, a local survey showed that 33.6% of high school students exhibited depressive symptoms [9]. Moreover, depression is a leading cause of illness and disability among adolescents globally and may lead to suicide [7].

While early generations frequently perceived bullying as a normal part of growing up [10], numerous studies demonstrated that school bullying victimization could result in severe academic, physical, mental, and psychosocial consequences [11], including depression [4–6]. Previous research considered being bullied a form of social defeat, which could induce stress and consequentially cause depression [12]. Additionally, bullying may cause neurobiological changes that contribute to mental health problems [13]. Empirical studies in both Western and Chinese societies demonstrated that bullying victimization was associated with depression among children and adolescents [6, 14–16]. Moreover, a systematic review concluded that there was strong evidence supporting the causal effect of bullying victimization on depression [17]. Also, bullying victimization contributed to 10.8% of the burden of depressive disorders among children and adolescents in Australia [18].

### The role of school connectedness

School connectedness was considered to exert a buffering effect on the negative health consequences of bullying victimization; specifically, as school connectedness increases, the negative effect of bullying victimization on health diminishes. According to the ecological systems theory [19], children’s development is influenced by multiple levels of environmental systems, with school being one of the most influential microenvironment systems. In schools, school connectedness, defined as a positive emotional connection between individuals, their schools, and the people in their schools [20], is a significant experience for children and a perceived support factor [20]. The stress-buffering model [21] indicates that social support may mitigate the adverse health effects of stress. School bullying victimization may lead to health issues due to stress [12]; thus, school connectedness could mitigate the negative health effects of school bullying victimization by providing social support to alleviate the stress. Empirical research among children and adolescents demonstrated that school connectedness was associated with better mental health, and the effect of bullying victimization on adjustment problems and mental health decreased as school connectedness increased [22–25]. However, a previous study showed contradictory results, suggesting that school connectedness may fail to protect the health and well-being of youth as peer bullying intensifies [26]. The above contradictory results might be due to different outcomes examined in these studies. For example, the previous study [26] on health and well-being used latent profile analysis to generate a 5-group outcome variable based on 11 health and well-being indicators. By contrast, other studies [22–25] examined different health or well-being indicators separately.

Additionally, school connectedness could be eroded by school bullying victimization, subsequently resulting in increased levels of depression. As pointed out by the social support deterioration model [27, 28], traumatic events may result in the perception of diminished social support, consequently jeopardizing health and well-being. In this sense, school bullying victimization, as a severely traumatic experience for school children, may lead victims to perceive a breakdown of reliable social support and diminished connections with others in their school, thereby exacerbating depression [29, 30]. Also, secure connectedness with others is essential to fulfill the need for relatedness, which is regarded as one of the basic psychological needs for human development [31]. Secure connectedness and relatedness are determined by not only individual factors but also social factors [31]. In this sense, bullying as a negative school social factor may damage victims’ connectedness with others, thereby jeopardizing their mental health. Moreover, bullying is related to social identity [32]. Specifically, bullying may cause victims to view themselves as out-group members, alienated from their school and the people in their school, thereby adversely affecting their mental health. Empirical research also provided evidence that bullying victimization could damage victims’ school connectedness [33, 34].

Taken together, school connectedness could be a moderator and a mediator in the association between school bullying victimization and mental health; however, few previous studies, if not none, examined the role of school connectedness as both a moderator and a mediator simultaneously in one study with the same outcome. Hence, little is known whether school connectedness plays both a moderating and a mediating role in the association between school bullying victimization and the same health outcome. Additionally, previous empirical studies regarding the role of school connectedness on the association between school bullying victimization and health outcomes mainly relied on cross-sectional designs, which were subject to confounding bias caused by unobserved factors.

### The present study

**Fig. 1:**
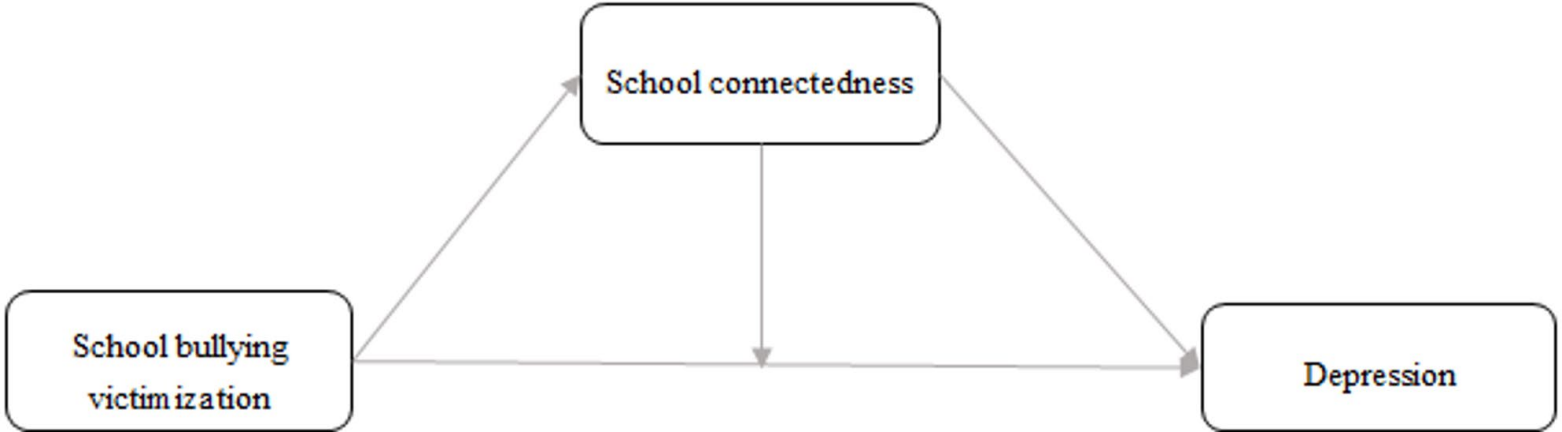
Analytical Framework

We examined the following among junior high school students in Hong Kong to fill the above research gaps: (1) the associations of school bullying victimization and school connectedness with depression, (2) the moderating effect of school connectedness on the association between school bullying victimization and depression, and (3) the mediating effect of school connectedness linking school bullying victimization to depression (Fig. 1). In doing so, the contribution of this study to previous literature is two-fold. First, focusing on the same outcome (i.e., depression) in this study, we could examine whether school connectedness played a moderating role, a mediating role, or both roles simultaneously in the association between school bullying victimization and this same health outcome. Second, based on two-wave follow-up data, we employed fixed-effects models to rule out the effect of all the observed and unobserved time-invariant confounders on the above associations. School bullying victimization in this research refers to school students’ experience of being targeted by other students in school using aggressive behaviors, including physical, verbal, social, threat, and sexual forms [35, 36].

## Methods

### Data source and participants

Our data are from a two-wave follow-up survey conducted among junior high school students (Grades 7–9) in Hong Kong. The duration between the two waves of survey was 9 months. A cluster random sampling method was employed to recruit participants from all three administrative areas in Hong Kong (namely, Hong Kong Island, Kowloon, and New Territories) in the survey. For the New Territories, it is further divided into New Territories East and New Territories West. Therefore, at the first stage of sampling, we divided Hong Kong into four areas (New Territories East, New Territories West, Hong Kong Island, and Kowloon). In each area, one school was randomly selected. At the second stage, one class from each grade level (i.e., Grades 7, 8, and 9) of the school was randomly selected. Then, all the students from a selected class were invited to participate in the survey. In Wave 1, a total of 392 students were surveyed, of whom 356 completed both the two waves of the survey, comprising the sample in this study. We compared the sample characteristics, the dependent variable, the independent variable, and the mediator in Wave 1 between those who were followed up and those who were lost to follow-up and did not find significant differences.

### Measurements

#### Health outcome: depression

The health outcome was depression, measured using seven items from the depression dimension of the Brief Symptom Rating Scale [37]. Each item was rated on a five-point Likert scale ranging from 0 (not at all) to 4 (extremely). We calculated the average score of the seven items for each participant in each wave as the outcome. Hence, the depression outcome was a score from 0 to 4, with a higher score indicating a higher level of depression. The Cronbach’s α coefficients for the seven items were 0.8575 and 0.9034 in Waves 1 and 2, respectively.

#### Exposure: school bullying victimization

The traditional Chinese version of the California School Climate and Safety Survey was adapted to measure the students’ school bullying victimization by other students since the beginning of the semester [36, 38]. Specifically, 13 items covering the five most common types of school victimization (namely, verbal, social, physical, sexual, and threats) were utilized, with each item rated on a five-point Likert scale from 1 (never) to 5 (seven times and above). We calculated the total score of the 13 items; hence, school bullying victimization ranged from 13 to 65, with a higher score indicating a higher level of victimization. The Cronbach’s α coefficients for the 13 items were 0.8997 and 0.8748 in Waves 1 and 2, respectively.

#### School connectedness as a potential mediator and moderator 

School connectedness was measured using five items from the National Longitudinal Study of Adolescent Health [39, 40]: (1) I feel close to people at this school; (2) I feel like I am part of this school; (3) I am happy to be at this school; (4) The teachers at this school treat students fairly; (5) I feel safe in my school. Each item was rated on a five-point Likert scale ranging from 1 (strongly disagree) to 5 (strongly agree). We calculated the total score of the five items; hence, school connectedness ranged from 5 to 25, with a higher score indicating a higher level of school connectedness. The Cronbach’s α coefficients of the five items were 0.7977 and 0.8683 in Waves 1 and 2, respectively.

#### Covariates

We included the following observed covariates: sex (male vs. female), grade (7, 8, or 9), parental marital status (married vs. divorced/separated/others), and subjective family economic status (poor/ordinal vs. comfort/rich). Sex and grade are time-invariant variables in this study.

### Statistical analysis

We conducted descriptive statistics and reported mean with standard deviation (SD) for continuous variables and percentages for categorical variables in Waves 1 and 2.

We employed fixed-effects linear regression models using the first-difference approach [41] to examine the associations between school bullying victimization, school connectedness, and depression. The fixed-effects models allowed us to eliminate confounding effects of all the observed (namely, sex and grade in this study) and unobserved time-invariant variables using the within-subject differences in dependent and independent variables. Additionally, we controlled parental marital status and subjective family economic status as time-variant variables in all the fixed-effects linear regression models. We further specified an interaction term between school bullying victimization and school connectedness in the fixed-effects model to examine the moderating/buffering effect of school connectedness on the association between school bullying victimization and depression.

To examine the mediating effect of school connectedness on the association between school bullying victimization and depression, we examined both the association between school bullying victimization and school connectedness and the association between school connectedness and depression using fixed-effects linear regression. When both the two associations were statistically significant, we would further conduct a mediation analysis using seemingly unrelated regression (SUR) with the first-difference approach. In other words, the mediation analysis examined whether the association between the change in school bullying victimization and the change in depression was mediated by the change in school connectedness.

Additionally, we performed multiple imputation by chained equations (MICE) to address missing data issue [42]. We generated 20 imputed datasets and conducted all the above regression models based on the imputed datasets.

We utilized Stata/MP software (version 14.2 and version 19.5) for all data analysis, and *p* < 0.05 was considered statistically significant. The survey was approved by the Survey and Behavioral Research Committee of the Chinese University of Hong Kong.

## Results

### Sample characteristics

We reported the sample characteristics without imputation in Table 1 and after imputation in Appendix Table A1. Among the participants, 58.29% were female students, and 32.87%, 33.43%, and 33.71% were in Grades 7, 8, and 9, respectively.

**Table 1:** Sample characteristics (*N* of participants=356)

	Wave 1	Wave 2
	Mean ± SD / Column %	Mean ± SD / Column %
Depression^a^ (0–4)	0.84 ± 0.80	0.80 ± 0.82
Sex^b^		
Female	58.29%	58.29%
Male	41.71%	41.71%
Grade^c^		
Grade 7	32.87%	32.87%
Grade 8	33.43%	33.43%
Grade 9	33.71%	33.71%
Parental marital status^d^		
Married	76.76%	76.22%
Divorced/separated/others	23.24%	23.78%
Family economic status^e^		
Poor/ordinal	71.38%	69.82%
Comfort/rich	28.62%	30.18%
School bullying victimization by peers^f^ (13–65)	17.52 ± 6.85	17.23 ± 7.02
School connectedness^g^ (5–25)	17.93 ± 3.89	17.82 ± 3.77

Missing data: ^a^ 56; ^b^ 12; ^c^ 0; ^d^ 57; ^e^ 59^; f^ 56; ^g^ 59

### Associations of school bullying victimization and school connectedness with depression

We further reported the associations between school bullying victimization, school connectedness, and depression based on fixed-effects linear regression models in Table 2 and based on pooled ordinal least regression (OLS) models in Appendix Table A2. Model 1 in Table 2 shows that a one-unit increase in school bullying victimization was associated with a 0.031-unit increase in depression (*β* = 0.031, 95% CI: 0.017, 0.045) after controlling sex, grade, parental marital status, family economic status, and unobserved time-invariant variables. In Model 2, we added school connectedness as an independent variable. Model 2 shows that a one-unit increase in school connectedness was associated with a 0.049-unit decrease in depression (*β* = -0.049, 95% CI: -0.078, -0.021) after controlling school bullying victimization, sex, grade, parental marital status, family economic status, and unobserved time-invariant variables. Additionally, the effect size of school bullying victimization on depression decreased in Model 2 compared with that in Model 1 (0.031 in Model 1 vs. 0.027 in Model 2) after adding school connectedness as an independent variable. Compared with results from pooled OLS models (Appendix Table A2 Model A1 and Model A2), the effect size of bullying victimization and school connectedness from fixed-effects models (Model 1 and Model 2) in Table 2 were smaller, indicating that without controlling for unobserved time-invariant variables, results from OLS models might overestimate the effects of bullying victimization and school connectedness on depression.

**Table 2:** Associations of school bullying victimization and school connectedness with depression (*N* of participants=356)

	Model 1: Depression	Model 2: Depression	Model 3: Depression	Model 4: School connectedness	
	β Coefficient (95% CI)	β Coefficient (95% CI)	β Coefficient (95% CI)		β Coefficient (95% CI)
School bullying victimization	0.031^***^ (0.017,0.045)	0.027^***^ (0.013,0.040)	0.025^***^ (0.012,0.038)		-0.080^**^ (-0.138,-0.021)
School connectedness	--	-0.049^**^ (-0.078,-0.021)	-0.048^**^ (-0.007,-0.018)		--
School bullying victimization × School connectedness	--	--	-0.002 (-0.005,0.001)		--

Time-variant control variables include parental marital status and family economic status. Time-invariant observed control variables include sex and grade

^*^ *p* < 0.05, ^**^ *p* < 0.01, ^***^ *p* < 0.001

### The buffering effect of school connectedness

In Model 3 of Table 2, we added the interaction term between school bullying victimization and school connectedness in the fixed-effects model. Model 3 shows that the interaction effect between school bullying victimization and school connectedness on depression was not statistically significant (*β* = -0.002, 95% CI: -0.005,0.001), indicating that we had no evidence to support the buffering effect of school connectedness on the association between school bullying victimization and depression. However, the traditional OLS model (Model A3 in Table A2) shows that the interaction effect between school bullying victimization and school connectedness was significant. The differences between the traditional OLS model (Model A3) and the fixed-effects model (Model 3) might also indicate that, without controlling for unobserved time-invariant variables, the interaction effect might be biased.

### The mediating effect of school connectedness

In Model 4 of Table 2, we examined the association between school bullying victimization and school connectedness after controlling sex, grade, parental marital status, family economic status, and unobserved time-invariant variables. Model 4 shows that a one-unit increase in school bullying victimization was associated with a 0.080-unit decrease in school connectedness (*β* = -0.080, 95% CI: -0.138, -0.021). Compared with Model A4 of Table A2, the effect size of school bullying victimization on school connectedness in Model 4 of Table 2 was smaller, suggesting that the OLS model might overestimate the effect of school bullying victimization on school connectedness.

**Fig. 2:**
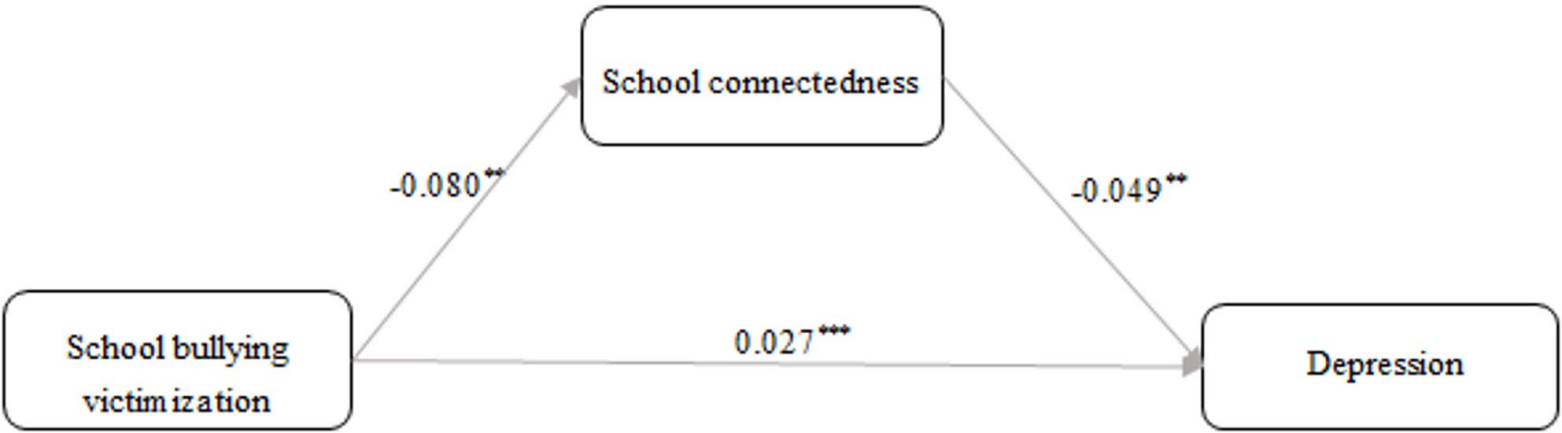
The mediating effect of school connectedness

Putting Model 2 together with Model 4, the results suggested that school connectedness could be a mediator between school bullying victimization and depression, as illustrated in Fig. 2. Specifically, school bullying victimization was associated with reduced school connectedness, and reduced school connectedness was further associated with increased depression.

Therefore, we further conducted a mediation analysis to examine the mediating effect of school connectedness on the association between school bullying victimization and depression. We reported the results of the mediation analysis in Table 3. It can be seen in Table 3 that a higher level of school bullying victimization was significantly associated with a higher level of depression through school connectedness (mediating effect: β = 0.004, 95% CI: 0.000, 0.008). Moreover, the mediating effect of school connectedness accounted for 12.86% of the association between school bullying victimization and depression after controlling sex, grade, parental marital status, family economic status, and unobserved time-invariant variables.

**Table 3:** The mediating effect of school connectedness on the association between school bullying victimization and depression

	Total effect on depression	Indirect effect via school connectedness	Mediation proportion
	β (95% CI)	β (95% CI)	
School bullying victimization	0.031^***^ (0.017,0.045)	0.004^*^ (0.000, 0.008)	12.86%

Time-variant control variables include parental marital status and family economic status. Time-invariant observed control variables include sex and grade

Results are estimated by “seemingly unrelated regression”

^*^ *p* < 0.05, ^**^ *p* < 0.01, ^***^ *p* < 0.001

### Additional analysis

Considering students from the same school were not independent of each other, cluster standard errors at the school level need to be used. In our study, as the number of schools (i.e., clusters) is small, we employed the wild cluster bootstrap inference to estimate the cluster standard errors at the school level while correcting the small number of clusters issue. We conducted the wild cluster bootstrap inference using the “wildbootstrap” command in Stata 19. We repeated the regression analysis in Table 2 using the wild cluster bootstrap inference and reported the results in Appendix Table A3 using unimputed data. Generally, the results in Appendix Table A3 were consistent with those in Table 2.

Additionally, using fixed-effects models, we examined the alternative direction among school bullying victimization, school connectedness, and depression; that is, whether depression as an independent variable was associated with school bullying victimization through school connectedness. We reported the results in Appendix Table A4. Generally, as school connectedness as an independent variable was not associated with school bullying victimization (Appendix Table A4 Model A10 and Model A13), school connectedness was not a mediator linking depression to school bullying victimization.

Furthermore, we removed the safety item from the school connectedness measurement and used the other four items only to measure school connectedness. We repeated the regression and mediation analyses in Tables 2 and 3 using the 4-item school connectedness measurement and reported the results in Appendix Table A5 and Table A6. Generally, the results were consistent with those in Tables 2 and 3; that is, school bullying victimization was associated with depression through school connectedness, while school connectedness did not buffer the effect of school bullying victimization on depression.

## Discussion and conclusions

To our knowledge, this is the first study to examine both the moderating and mediating roles of school connectedness in the association between school bullying victimization and depression among junior high school students in Hong Kong. We employed fixed-effects models to eliminate the confounding effects of all observed and unobserved time-invariant variables on the association between school bullying victimization and depression. After taking into account the confounding effect of sex, grade, parental marital status, family economic status, and unobserved time-invariant factors, we found that school bullying victimization was associated with a higher level of depression, while school connectedness was associated with a lower level of depression. Moreover, school bullying victimization was associated with greater depression by eroding students’ school connectedness. However, we did not find evidence for the moderating/buffering effect of school connectedness on the association between school bullying victimization and depression.

### The association between school bullying victimization and depression

Our finding that school bullying victimization was associated with greater depression among junior high school students in Hong Kong is consistent with those of previous studies in Western and other Chinese societies [4–6, 16, 43]. A systematic review [17] interpreted the association between bullying victimization and depression as causal using the Hill criteria [44]. Our study further contributed to the evidence of the causal effects in the sense of eliminating time-invariant confounding factors. Specifically, the association of bullying victimization with depression could be affected by unobserved individual characteristics. Previous studies employed twin data to partially control the effects of genetics on the association of bullying victimization with psychotic symptoms and internalizing problems [45, 46], but not depression. By contrast, we took another approach, namely, the fixed-effects models based on follow-up data, to address the confounding effect of unobserved time-stable individual characteristics, which may include a component of biological predispositions, on the association between school bullying victimization and depression, as fixed-effects models exclusively employ within-subject information. In other words, the fixed-effects models enabled a comparison of participants with themselves, thereby controlling for unobserved time-stable individual characteristics that remain largely constant for the same participant surveyed twice with a 9-month interval. Additionally, fixed-effects models can control for other time-invariant but unmeasured variables. For example, previous life experiences, such as health issues, may contribute to both bullying victimization and depression. Researchers cannot collect data on all of the previous life experiences. In this case, the fixed-effects model can eliminate potential confounding effects of these previous life experiences because for each person, they remain unchanged in subsequent life.

Moreover, compared with twin studies, our study, which employed fixed-effects models with a representative sample, may be more representative as non-twins (most of the population) were included. Hence, taking our study together with previous studies that demonstrated the temporality and biological plausibility of the association between school bullying victimization and depression [17], we are more confident in regarding the association as causal.

### The mediating effect of school connectedness

In this study, we found that increased school bullying victimization was associated with increased depression through reduced school connectedness. Previous studies in the broader fields of social capital, social cohesion, and social connectedness predominantly regarded social interactions as the basis of beneficial social relationships that enhance health [47–53]. However, social interactions may also involve conflicts and adverse social relationships, including school bullying. School bullying victimization stemming from conflictual social interaction could cause social relationship deterioration in school, thereby undermining school connectedness.

Also, bullying victimization may not only affect the social relationship between the victims and the perpetrators but also the victims’ perception of the general social environment in the school. A previous study demonstrated that bullying victimization was associated with lower trust [54]. In this sense, bullying victimization may diminish overall trust in the school, consequently decreasing the school connectedness of students who experienced a higher level of school bullying victimization. Additionally, bullying victimization may cause social withdrawal as a defensive coping strategy [55]. A previous study indicated that social withdrawal could reduce intimacy [56]. Therefore, bullying victimization could reduce school connectedness by inducing social withdrawal and subsequent decreased intimacy in school.

The diminished school connectedness may further induce adverse health conditions, including depression, which is consistent with findings of previous research on social disconnectedness and mental health [57, 58]. One reason for the negative association between school connectedness and depression is that connectedness is one of the basic human needs, and the deprivation of the basic need may lead to ill health [59–62]. Diminished school connectedness could also result in perceived isolation and loneliness, which could further cause depression [57]. Additionally, school connectedness might increase the probability of seeking help. A previous study found that school connectedness was associated with a higher likelihood of help-seeking behavior for suicide among college students [63]. Hence, it might also be the case that school connectedness could enhance junior high school students’ help-seeking behaviors for mental health. School connectedness may also enhance help-seeking behaviors for other challenges, thereby reducing the level of depression. Conversely, reduced school connectedness may inhibit students from seeking help, thereby increasing the level of depression.

Additionally, it should be noted that school connectedness accounted for 12.86% of the association between school bullying victimization and depression. In other words, approximately 87% of the association may be attributed to other factors or the direct effect of bullying victimization on depression. Therefore, more studies are needed to understand the mechanisms of the effect of bullying victimization on depression.

### The moderating effect of school connectedness

We did not observe a moderating effect of school connectedness on the association between school bullying victimization and depression. One reason could be our utilization of fixed-effect models with two-wave data. The fixed-effects models only use within-subject information; hence, they may result in insufficient within-subject variation of school connectedness and school bullying victimization between two waves in our data to identify a statistically significant interaction effect. Future studies with more waves of follow-up data and a larger sample size are needed to examine the moderating effect of school connectedness on the association between school bullying victimization and depression.

### Limitations

There are several limitations in this study. First, the fixed-effects models could not rule out the effects of time-variant confounders. Hence, the results may be subject to confounding bias caused by time-variant variables. Second, we could not rule out the possibility of reverse causality in this study because, in the fixed-effects models, the changes in bullying victimization, school connectedness, and depression occurred contemporaneously. In other words, the fixed-effects models in our study were not used to estimate the directional temporal effects across waves. Hence, the results in our study might not exactly establish causality. Additionally, our sample size may not be large enough, which might have limited the generalizability of the results and hampered us from detecting statistically significant effects, particularly regarding the interaction effect between school connectedness and school bullying victimization on depression. Also, the sample was from Hong Kong and covered only junior high school students (i.e., Grades 7, 8, and 9), which may have limited the generalizability of the results to adolescents in other educational levels and other societies.

### Conclusions

School bullying victimization could erode school connectedness, which in turn increased victims’ depression level. Therefore, schools need to help students who experienced bullying reestablish their connectedness with the school and its members to alleviate the health effects of bullying victimization. Interventions to promote the health of students who experienced bullying need to consider the overall school environment in addition to the individual factors that contribute to health.

## Supplementary Information

Supplementary Material 1.

## Data Availability

The datasets during and/or analysed during the current study available from the corresponding author on reasonable request.
