# Supplementary material for: School bullying victimization, depression, and the role of school connectedness among junior high school students in Hong Kong: evidence from fixed-effects models
Source: BMC Psychol. 2026 Apr 25;14:845. doi: 10.1186/s40359-026-04614-2 (PMC13248397; doi:10.1186/s40359-026-04614-2)
Supplement: Supplementary file 1 — Supplementary Material 1. [file 40359_2026_4614_MOESM1_ESM.docx]

**Appendix: Supplementary data**

**Table A1. Sample characteristics after imputation (N of participants=356)**

|  | Wave 1 | Wave 2 |
| --- | --- | --- |
|  | Mean±SD / Column % | Mean±SD / Column % |
| **Depression^a^ (0-4)** | 0.84±0.80 | 0.80±0.82 |
| **Gender^b^** |  |  |
| Female | 58.15% | 58.15% |
| Male | 41.85% | 41.85% |
| **Grade^c^** |  |  |
| Grade 7 | 32.87% | 32.87% |
| Grade 8 | 33.43% | 33.43% |
| Grade 9 | 33.71% | 33.71% |
| **Parental marital status^d^** |  |  |
| Married | 76.88% | 76.50% |
| Divorced/separated/others | 23.12% | 23.50% |
| **Family economic status^e^** |  |  |
| Poor/ordinal | 71.11% | 69.59% |
| Comfort/rich | 28.89% | 30.41% |
| **School bullying victimization by peers^f^ (13-65)** | 17.48±6.88 | 17.18±7.03 |
| **School connectedness^g^ (5-25)** | 17.93±3.89 | 17.84±3.79 |

Missing data: ^a^ 56; ^b^ 12; ^c^ 0; ^d^ 57; ^e^ 59^; f^ 56; ^g^ 59

**Table A2. Pooled OLS: Associations of school bullying victimization and school connectedness with depression (N of participants=356)**

|  | Model A1: Depression | Model A2: Depression | Model A3: Depression | Model A4: School connectedness | |
| --- | --- | --- | --- | --- | --- |
|  | *β* Coefficient (95% CI) | *β* Coefficient (95% CI) | *β* Coefficient (95% CI) | | *β* Coefficient (95% CI) |
| School bullying victimization | 0.044^***^ (0.036,0.053) | 0.034^***^ (0.026,0.042) | 0.060^***^ (0.036,0.084) | | -0.155^***^ (-0.197,-0.113) |
| School connectedness | -- | -0.067^***^ (-0.082,-0.052) | -0.036^*^ (-0.067,-0.005) | | -- |
| School bullying victimization × School connectedness | -- | -- | -0.002^*^ (-0.003,-0.000) | | -- |

^*^ *p* < 0.05, ^**^ *p* < 0.01, ^***^ *p* < 0.001

Control variables include gender, grade, parental marital status, family economic status, and survey wave.

**Table A3. Associations of school bullying victimization and school connectedness with depression: Fixed-effects models with wild cluster bootstrap inference on unimputed data (N of participants=299)**

|  | Model A5: Depression | Model A6: Depression | Model A7: Depression | Model A8: School connectedness | |
| --- | --- | --- | --- | --- | --- |
|  | *β* Coefficient (95% CI) | *β* Coefficient (95% CI) | *β* Coefficient (95% CI) | | *β* Coefficient (95% CI) |
| School bullying victimization | 0.027^*^  (0.004, 0.161) | 0.025^*^  (0.002, 0.159) | 0.025^*^  (0.001, 0.154) | | -0.051^*^  (-0.066, -0.008) |
| School connectedness | -- | -0.032^*^  (-0.059, -0.011) | -0.032^*^  (-0.060, -0.009) | | -- |
| School bullying victimization × School connectedness | -- | -- | -0.0003 (-0.027,0.022) | | -- |

^*^ *p* < 0.05, ^**^ *p* < 0.01, ^***^ *p* < 0.001

Time-variant control variables include parental marital status and family economic status. Time-invariant observed control variables include gender and grade.

Wild bootstrap CIs with Webb weights clustering at the school level were used to correct the small numbers of clusters issue.

**Table A4. Associations of depression and school connectedness with school bullying victimization**

|  | Imputed | | | |  | Unimputed with wild cluster bootstrap inference^$^ | | | | |  |
| --- | --- | --- | --- | --- | --- | --- | --- | --- | --- | --- | --- |
|  | Model A9: School bullying victimization | Model A10: School bullying victimization | Model A11: School connectedness | |  | Model A12: School bullying victimization | Model A13: School bullying victimization | | Model A14: School connectedness | |  |
|  | *β* Coefficient (95% CI) | *β* Coefficient (95% CI) | *β* Coefficient (95% CI) |  | | *β* Coefficient (95% CI) | | *β* Coefficient (95% CI) | | *β* Coefficient (95% CI) | |
| Depression (0-4) | 3.380^***^ (2.011,4.749) | 3.090^***^ (1.625,4.556) | -1.275^***^ (-1.896,-0.654) |  | | 2.555  (-4.260, 5.278) | | 2.444  (-0.566, 5.193) | | -0.826^*^  (-1.164, -.098) | |
| School connectedness | -- | -0.227 (-0.544,0.090) | -- |  | | -- | | -0.134  (-0.607, 0.302) | | -- | |
| N of participants | 356 | 356 | 356 |  | | 299 | | 299 | | 299 | |

^*^ *p* < 0.05, ^**^ *p* < 0.01, ^***^ *p* < 0.001

Time-variant control variables include parental marital status and family economic status. Time-invariant observed control variables include gender and grade.

^$^Wild bootstrap CIs with Webb weights clustering at the school level were used to correct the small numbers of clusters issue.

**Table A5. Associations of school bullying victimization and school connectedness with depression: Fixed-effects models, with no safety item in school connectedness**

|  | Imputed (N=356) | | | | |  | Unimputed with wild cluster bootstrap inference^$^ (N=302) | | | |
| --- | --- | --- | --- | --- | --- | --- | --- | --- | --- | --- |
|  | Model A15: Depression | Model A16: Depression | Model A17: Depression | Model A18: School connectedness | |  | Model A19: Depression | Model A20: Depression | Model A21: Depression | Model A22: School connectedness |
|  | *β* Coefficient (95% CI) | *β* Coefficient (95% CI) | *β* Coefficient (95% CI) | | *β* Coefficient (95% CI) |  | *β* Coefficient (95% CI) | *β* Coefficient (95% CI) | *β* Coefficient (95% CI) | *β* Coefficient (95% CI) |
| School bullying victimization | 0.031^***^ (0.019,0.042) | 0.026^***^ (0.015,0.037) | 0.025^***^ (0.014,0.036) | | -0.074^**^  (-0.121,-0.028) |  | 0.027^*^  (0.004, 0.159) | 0.024^*^  (0.002, 0.157) | .024  (-0.103, 0.159) | -.046^*^  (-0.079, -0.005) |
| School connectedness | -- | -0.060^***^ (-0.092,-0.028) | -0.058^***^ (-0.089,-0.026) | | -- |  | -- | -0.041^*^  (-0.064, -0.028) | -0.039^*^  (-0.067, -0.025) | -- |
| School bullying victimization × School connectedness | -- | -- | -0.001 (-0.005,0.002) | | -- |  | -- | -- | .002  (-0.052, 0.031) | -- |

^*^ *p* < 0.05, ^**^ *p* < 0.01, ^***^ *p* < 0.001

Time-variant control variables include parental marital status and family economic status. Time-invariant observed control variables include gender and grade.

^$^Wild bootstrap CIs with Webb weights clustering at the school level were used to correct the small numbers of clusters issue.

**Table A6. The mediating effect of school connectedness on the association between school bullying victimization and depression, with no safety item in school connectedness (N=356)**

|  | Total effect on depression | Indirect effect via school connectedness | Mediation proportion |
| --- | --- | --- | --- |
|  | *β* (95% CI) | *β* (95% CI) |  |
| School bullying victimization | 0.031^***^ (0.019,0.042) | 0.004^*^  (0.001, 0.008) | 14.56% |

^*^ *p* < 0.05, ^**^ *p* < 0.01, ^***^ *p* < 0.001

Time-variant control variables include parental marital status and family economic status. Time-invariant observed control variables include gender and grade.

Results are estimated by “seemingly unrelated regression.”
